# Supplementary material for: Not All Weight Loss Is Equal: Divergent Patterns and Prognostic Roles in Head and Neck Cancer Versus High-Grade B-Cell Lymphoma
Source: Nutrients. 2025 Jul 31;17(15):2530. doi: 10.3390/nu17152530 (PMC12348341; doi:10.3390/nu17152530)
Supplement: Supplementary file 1 [file nutrients-17-02530-s001.zip › nutrients-3760293-supplementary.pdf]

nutrients

MDPI

Supplementary Table S1. Δweight over time. HNC – head neck cancer, HGBCL – high grade b-cell lymphoma, SD – standard deviation

	HNC + HGBCL	HNC	HGBCL
**T_1 (3 months)**			
Δweight, Stable weight [%], mean +/- SD	-0.94 +/- 2.48	-1.12 +/_2.46	-0.84 +/- 2.50
Δweight, Weight loss [%], mean +/- SD	-10.05 +/- 5.72	-10.80 +/- 4.24	-9.51 +/- 6.53
Δweight, Weight gain [%], mean +/- SD	7.97 +/- 1.80	n/A	7.97 +/- 1.80
**T_2 (6 months)**			
Δweight, Stable weight [%], mean +/- SD	-0.38 +/- 2.87	-0.33 +/- 2.81	-0.42 +/-2.91
Δweight, Weight loss [%], mean +/- SD	-12.14 +/- 5.07	-12.23 +/- 5.27	-12.05 +/- 4.82
Δweight, Weight gain [%], mean +/- SD	9.58 +/- 4.65	n/A	9.58 +/- 4.65
**T_3 (9 months)**			
Δweight, Stable weight [%], mean +/- SD	0.25 +/- 2.91	0.31 +/- 2.75	-0.49 +/- 2.82
Δweight, Weight loss [%], mean +/- SD	12.70 +/- 5.67	-12.70 +/- 5.41	-12.70 +/- 6.04
Δweight, Weight gain [%], mean +/- SD	10.30 +/- 4.61	6.27 +/- 0.32	11.46 +/- 4.62
**T_4 (12 months)**			
Δweight, Stable weight [%], mean +/- SD	0.00 +/- 0.00	-0.37 +/- 2.61	-0.96 +/- 2.52
Δweight, Weight loss [%], mean +/- SD	-13.92 +/.7.07	-15.01 +/- 7.25	-12.00 +/- 6.29
Δweight, Weight gain [%], mean +/- SD	8.00 +/-1.67	7.35 +/- 1.45	8.15 +/- 1.68

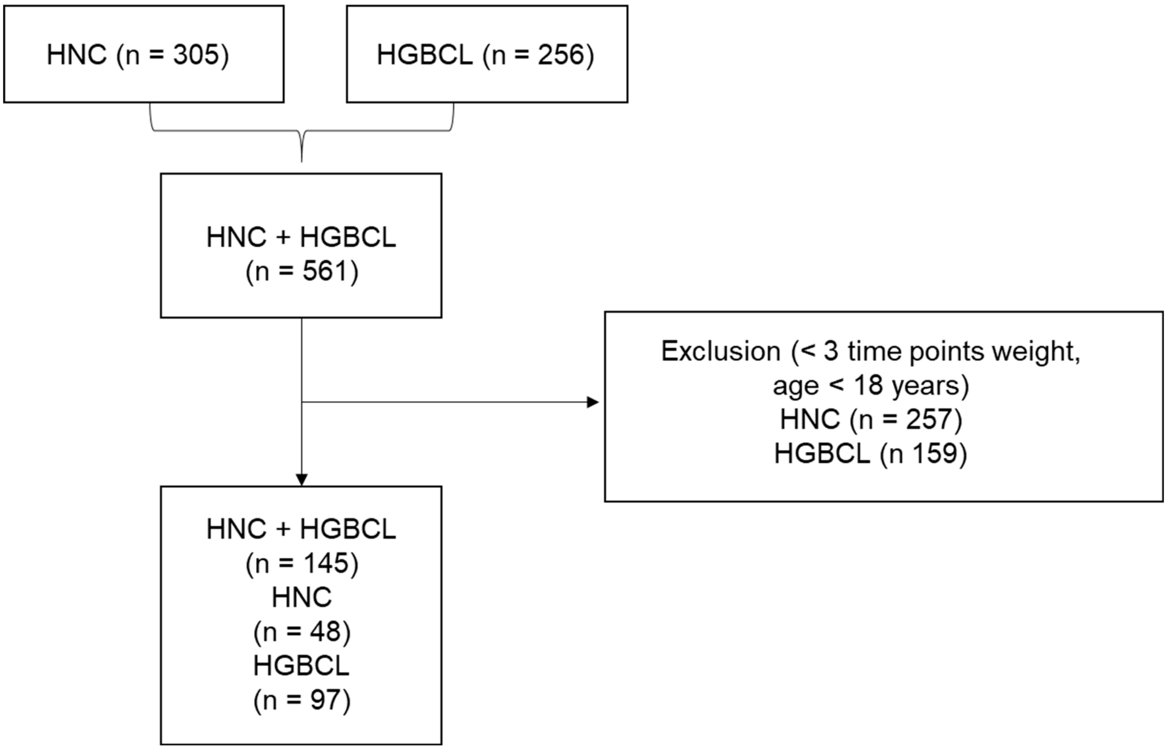

***Supplementary Figure S1.** Flow chart depicting included cases of head heck (HNC) and high grade b-cell lymphoma (HGBCL) patients.*
